# Integrated metabolomics and genomics analysis provides new insights into the fiber elongation process in Ligon lintless-2 mutant cotton (*Gossypium hirsutum* L.)

**DOI:** 10.1186/1471-2164-14-155

**Published:** 2013-03-07

**Authors:** Marina Naoumkina, Doug J Hinchliffe, Rickie B Turley, John M Bland, David D Fang

**Affiliations:** 1 Cotton Fiber Bioscience Research Unit, USDA-ARS, Southern Regional Research Center, New Orleans, LA 70124, USA; 2 Cotton Chemistry & Utilization Research Unit, USDA-ARS, Southern Regional Research Center, New Orleans, LA, 70124, USA; 3 Crop Genetics Research Unit, USDA-ARS, Mid South Area, Stoneville, MS, 38772, USA; 4 Food Processing and Sensory Quality Research Unit, USDA-ARS, Southern Regional Research Center, New Orleans, LA, 70124, USA

**Keywords:** Cotton, *Li*_*2*_ mutation, Fiber, Elongation, Metabolomics, Gamma-aminobutyric acid (GABA)

## Abstract

**Background:**

The length of cotton fiber is an important agronomic trait characteristic that directly affects the quality of yarn and fabric. The cotton (*Gossypium hirsutum* L.) fiber mutation, Ligon lintless-2, is controlled by a single dominant gene (*Li*_*2*_) and results in extremely shortened lint fibers on mature seeds with no visible pleiotropic effects on vegetative growth and development. The *Li*_*2*_ mutant phenotype provides an ideal model system to study fiber elongation. To understand metabolic processes involved in cotton fiber elongation, changes in metabolites and transcripts in the *Li*_*2*_ mutant fibers were compared to wild-type fibers during development.

**Results:**

Principal component analysis of metabolites from GC-MS data separated *Li*_*2*_ mutant fiber samples from WT fiber samples at the WT elongation stage, indicating that the *Li*_*2*_ mutation altered the metabolome of the mutant fibers. The observed alterations in the *Li*_*2*_ metabolome included significant reductions in the levels of detected free sugars, sugar alcohols, sugar acids, and sugar phosphates. Biological processes associated with carbohydrate biosynthesis, cell wall loosening, and cytoskeleton were also down-regulated in *Li*_*2*_ fibers. Gamma-aminobutyric acid, known as a signaling factor in many organisms, was significantly elevated in mutant fibers. Higher accumulation of 2-ketoglutarate, succinate, and malate suggested higher nitrate assimilation in the *Li*_*2*_ line. Transcriptional activation of genes involved in nitrogen compound metabolism along with changes in the levels of nitrogen transport amino acids suggested re-direction of carbon flow into nitrogen metabolism in *Li*_*2*_ mutant fibers.

**Conclusions:**

This report provides the first comprehensive analysis of metabolite and transcript changes in response to the *Li*_*2*_ mutation in elongating fibers. A number of factors associated with cell elongation found in this study will facilitate further research in understanding metabolic processes of cotton fiber elongation.

## Background

Cotton fiber is the most prevalent natural raw material used in the textile industry. Cotton seed fibers are highly elongated single-celled trichomes that differentiate from the outer epidermis of the ovule. The seeds of the cultivated cotton produce two types of fibers: short or fuzz hairs that have little commercial value; and long or lint hairs that are removed from the seeds during the ginning process and used for yarn production by the textile industry. Only 25–30% of epidermal cells differentiate into lint fiber [1]. Fiber development occurs in four distinct, but overlapping stages – initiation, elongation, secondary cell wall synthesis, and maturation [1-3]. The initiation stage starts from - 3 day of anthesis (DOA) to 3 days post-anthesis (DPA), and is followed by the fiber elongation stage. The lint fiber cells elongate for about 27–39 DPA and the secondary cell wall is formed from 17 to 53 DPA depending of the cotton species, cultivar and environment [4]. Cotton fiber initiation stage acts as a developmental switch to determine the number of fibers on each ovule. The extent of the elongation period determines fiber length, which ranges from 25–40 mm, while the extent of secondary wall thickening determines fiber diameter. Fiber properties are largely quantitative traits and environment conditions can determine whether the fibers reach the genetic potential of the cotton cultivar. Cotton of superior quality and value typically consists of long, fine, and strong fiber. The length of the fiber is one of the most important characteristics and affects spinning efficiency and the quality of the resulting yarn [1]. Competition with synthetic fibers has forced cotton industry to invest heavily in research to develop higher-quality fibers; however, one of the major limitations in genetic improvement of fiber is the lack of information at the molecular level regarding genes and regulatory elements that control fiber development. Elucidating the cellular and molecular basis of fiber elongation could identify potential targets for genetic manipulation of fiber length.

**Figure 1:**
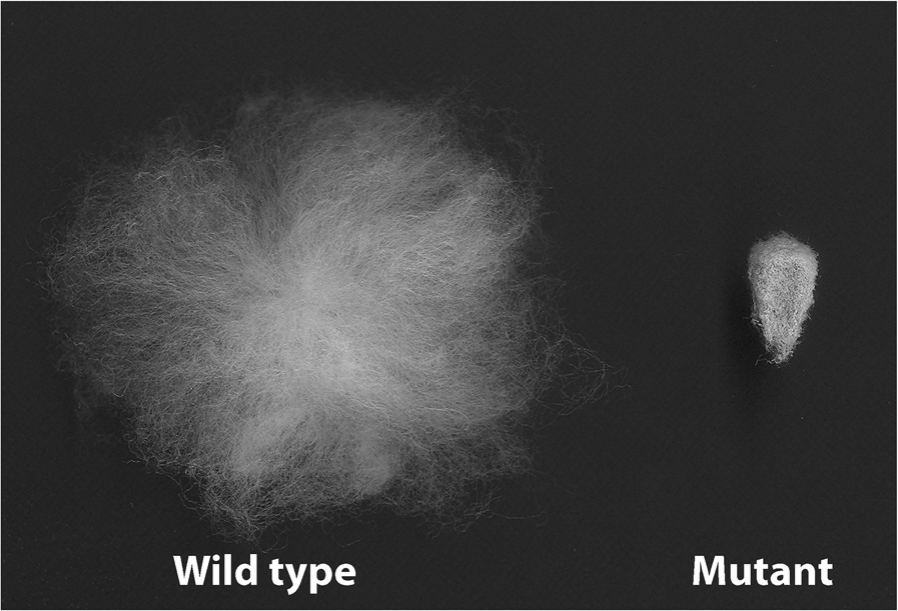
**Comparison of mature *Li***_***2 ***_**mutant and WT seeds with fibers.**

Genetic mutants are useful tools for studying gene function. In cotton several fiber-related mutants were discovered, from which Ligon lintless-1 (*Li*_*1*_) and Ligon lintless-2 (*Li*_*2*_) were reported to be monogenic and dominant, resulting in an extreme reduction in the length of lint fiber to approximately 6 mm on mature seeds [5,6]. It has been determined that *Li*_*1*_ associated with chromosome 22, whereas *Li*_*2*_ with chromosome 18 [7-9]. Cytological studies did not reveal much difference in seed fiber initiation between mutants and their near-isogenic lines suggesting the effects of the mutation occurs later in development during the elongation stage [9,10]. Kohel *et al*. observed restricted and similar fiber elongation pattern for the mutant lines, comparing *Li*_*1*_ and *Li*_*2*_ with TM-1 in a fiber developmental study [11]. Therefore, in a near-isogenic state with a wild-type (WT), these mutants represent good model system to study fiber elongation. Unlike the stunted and deformed vegetative morphology of *Li*_*1*_ plants [5], *Li*_*2*_ has normal vegetative growth, and the phenotype of the seed cotton is similar to *Li*_*1*_. Also dry weight of *Li*_*2*_ developing fiber was reported to be significantly lighter then *Li*_*1*_ that was attributed to difference in secondary wall development between the two mutants [11]. Our laboratory selected the *Li*_*2*_ mutant as a model system to study fiber elongation for the reasons mentioned above. An *Li*_*2*_ mutant cotton line in a near-isogenic state with the Upland cotton variety DP5690 was developed in a backcross program at Stoneville, MS [10]. Morphological evaluation by scanning electron microscopy revealed no visible differences in the appearances of ovules and fibers from *Li*_*2*_ mutant and WT near-isogenic lines (NILs) during initiation and early elongation up to 5 DPA [10]. Comparison of *Li*_*2*_ mutant and WT seeds with fibers at maturity is shown in Figure 1. In a previous report, an expressed sequence tag- simple sequence repeat (EST-SSR) marker with complete linkage to the *Li*_*2*_ genetic locus was identified using combined functional and structural genomics; and large-scale transcriptome analysis revealed changes in reactive oxygen species homeostasis and cytokinin regulation in *Li*_*2*_ mutant fibers compared to WT fibers [10].

Much attention has focused on transcriptome analysis to study fiber-related mutants [10,12-14], whereas biochemical analysis of the levels of low molecular weight compounds has been largely overlooked. The metabolome can be viewed as the end products of gene expression, and the measurements of large numbers of cellular metabolites provide a high resolution biochemical phenotype of an organism [15]. The phenotype can also be characterized by transcriptome or proteome analysis. However, mRNA levels do not always correlate with protein levels [16], and changes in profiling of transcripts or proteins may not always lead to alterations in the metabolic phenotype. Also, the majority of transcript and protein annotations are currently predicted based on sequence or structural motifs similarity and these annotations often offer limited information since many of these putative enzymes could be involved in a large number of different reactions. Metabolomics has the ability to reveal that the accumulated enzyme is more specifically related to specific biochemical reaction. Therefore, integrated approaches such as combined transcript, protein, and metabolite profiling offer greater opportunities for discovery and understanding of biological processes.

Gas chromatography–mass spectrometry (GC-MS) was previously applied to examine the effects of genetic and environmental manipulations [17]. GC-MS is currently the most developed of the available analytical tools and the growth of this technology offers the opportunity to view the effect of a single mutation on metabolism on a larger scale than previously possible. The goals of this study were to identify metabolic and transcript responses associated with fiber elongation using *Li*_*2*_ NILs. Significant changes in the relative abundance of multiple identified metabolites were observed between NILs which are the result of genetic reprogramming of primary metabolism in response to *Li*_*2*_ mutation. These results will facilitate future research in understanding metabolic processes controlling fiber elongation.

## Methods

### Plant materials

Two NILs of *Li*_*2*_ Upland cottons (*Gossypium hirsutum* L.) were developed in a backcross program at Stoneville, MS in field and greenhouse environments [10]. Growth conditions, greenhouse experimental design, and strategy of pooling samples were previously described [10]. A total of 72 mutant *Li*_*2*_*Li*_*2*_ plants and 72 WT *li*_*2*_*li*_*2*_ plants were used for samples collection. Cotton bolls were harvested at the following time-points during development: -3 day of anthesis (DOA), DOA, 1, 3, 5, 8, 12, 16, and 20 days post anthesis (DPA). Harvested bolls were placed immediately on ice and transported to the laboratory where they were dissected on ice, frozen in liquid nitrogen and stored at −80°C.

### SSR marker analysis

The *Li*_*2*_ parental NILs of the two mutant and WT populations were analyzed using SSR markers to determine their genetic similarity. Young leaves were collected from each one of the NIL parental line plants and total DNA was extracted from fresh leaves using 2.0% hexadecyltrimethylammonium bromide [18]. DNA was purified using Omega EZNA^®^ DNA isolation column (Omega Bio-Tek, Norcross, GA). To estimate the genetic similarity of the *Li*_*2*_ parental NILs, 1349 SSR markers were randomly chosen without any knowledge of their mapping positions. The SSR marker analysis was conducted as previously described [19].

### RNA isolation, RT-qPCR and microarray

Cotton fibers were isolated from developing ovules using a glass bead shearing technique to separate fibers from the ovules [20]. Total RNA was isolated from detached fibers using the Sigma Spectrum™ Plant Total RNA Kit (Sigma-Aldrich, St. Louis, MO) with the optional on column DNase1 digestion according to the manufacturer’s protocol. The concentration of each RNA sample was determined using a NanoDrop 2000 spectrophotometer (NanoDrop Technologies Inc., Wilmington, DE). The RNA quality for each sample was determined by RNA integrity number (RIN) using an Agilent Bioanalyzer 2100 and the RNA 6000 Nano Kit Chip (Agilent Technologies Inc., Santa Clara, CA) with 250 ng of total RNA per sample.

The experimental procedures and data analysis related to RT-qPCR were performed according to the Minimum Information for Publication of Quantitative Real-Time PCR Experiments (MIQE) guidelines [21]. Nine fiber developmental time-points mentioned above were used for RT-qPCR analyses. The detail description of reverse transcription, qPCR and calculation reported before [10]. Primers are available online in Additional file 1.

The Affymetrix GeneChip ^®^ Cotton Genome Array (Affymetrix Inc., Santa Clara, CA), containing 21,854 probe sets from four cotton species (*G. arboreum, G. barbadense, G. hirsutum, and G. raimondii*), was utilized for microarray experiment. Labeling, hybridization and data processing were performed according to standardized Affymetrix protocols. RNA from three developmental time-points (0, 8, and 12 DPA) with two biological replicates from *Li*_*2*_ mutant and WT fibers were used for microarray study. Procedures for data normalization and assessment of statistically and biologically significant genes were performed as described previously [22]. The Affymetrix microarray dataset was deposited in the ArrayExpress database with the accession number E-MEXP-3306.

### Sample processing, extraction and GC/MS metabolite analysis

Whole ovules for time-points at −3 DOA, DOA and 1 DPA were ground in liquid nitrogen and processed for freeze lyophilization; fiber tissue for time-points at 3, 5, 8, 12, 16 and 20 DPA was collected by shaking frozen ovules and processed for freeze lyophilization. The dried tissue was stored at −80°C until extraction. A 6.0 mg of dried tissue was weighed into 4 ml glass vial. The extraction method used in this study is an adaptation of previously reported method developed and optimized for Arabidopsis leaves [23]. Solvent containing methanol:chloroform:water (3:1:1) was used for extraction. Samples were extracted by shaking for 2 hours at room temperature with 1 ml of cold solvent contained internal standard 0.2 mg/ml ribitol. After centrifugation for 30 min at 3000 g 400 μl of the supernatant was transferred to new glass vial and dried overnight in speedvac. Dry samples were re-suspended in 30 μl pyridine with 2% methoxyamine HCl (Pierce, Rockford, IL) and incubated for 30 min at 50°C. Metabolites were then derivatized with 70 μl of MSTFA+1% TMCS (Pierce, Rockford, IL) for 1 h at 50°C. The samples were equilibrated to room temperature, transferred to a 250 μl glass insert and analyzed using an Agilent 6890 GC coupled to a 5973 MSD, scanning from *m/z* 40–550. Samples (2 μl) were injected with an Agilent 7683 autosampler into the GC inlet held at 270°C with a split ratio of 25:1. Separation was achieved on a fast GC column (MACH DB-5MS), 20m × 0.18mm × 0.18μm (Gerstel, Baltimore, MD, USA), temperature programmed for 60°C, held for 1 min, then ramped at 50°C/min to 310°C, and held for 4 min. The GC was pressure ramp programmed for 21.7 psi, held for 1 min, then ramped at 4.48 psi/min to 44.1 psi, and held for 4 min, to keep a constant flow of 1.0 ml/min helium. The column outlet was pressurized to 4 psi with a QuickSwap (Gerstel). The GC oven and MSD transfer line were held at 280°C.

### GC/MS data processing and analysis

Chromatograms were acquired with ChemStation software (Agilent, Santa Clara, CA, USA). Initial processing and export of the processed chromatograms into a *.cdf file interchange format were performed within the ChemStation software. Retention index (RI) calibration and mass spectral deconvolution were performed by AMDIS (Automated Mass Spectral Deconvolution and Identification System, National Institute of Standards and Technology, Gaithersburg, MD, USA) with the settings: adjacent peak subtraction = 2, medium resolution, high sensitivity, and high shape requirements. Identification was conducted using internal standards for RI. GC/MS peaks were annotated performing search against of freely available MSRI libraries [The Golm Metabolome Database http://csbdb.mpimp-golm.mpg.de/csbdb/dload/dl_msri.html[24]] matching mass spectra and retention index. Then, multiple raw data files were organized into one data matrix for further statistical interrogation using MET-IDEA software [Metabolomics Ion-based Data Extraction Algorithm, [25]]. Peak areas were normalized by dividing each peak area value by the area of the internal standard for a specific sample. Correlation and principal component analysis were performed on normalized datasets using JMP genomics 5 (SAS, Cary, NC, USA). Statistical analysis of 2way ANOVA (mutation and time as variables) was performed with JMP genomics 5 as well. Normalized GC-MS metabolite profiling data is provided in Additional file 2.

## Results

### Genetic similarity of the *Li*_*2*_ NILs

The development of the *Li*_*2*_ NILs utilized in this study were previously described and SSR markers specific to chromosomes 13 and 18 were selected to screen for polymorphisms in the *Li*_*2*_ parental NILs [10]. To further justify the use of the *Li*_*2*_ NILs and subsequent segregating populations as a model system to study cotton fiber elongation, the genetic similarity between the *Li*_*2*_ parental NILs was determined using SSR markers that were distributed across the entire Upland cotton genome. The marker analysis that was conducted utilizing 1,349 randomly chosen SSR markers revealed a total of 76 markers that were polymorphic between the *Li*_*2*_ parental NILs. Based on the ratio of polymorphic to non-polymorphic SSR markers, the genetic similarity of the NILs was estimated to be 94.4%.

### Global metabolic trends

**Figure 2:**
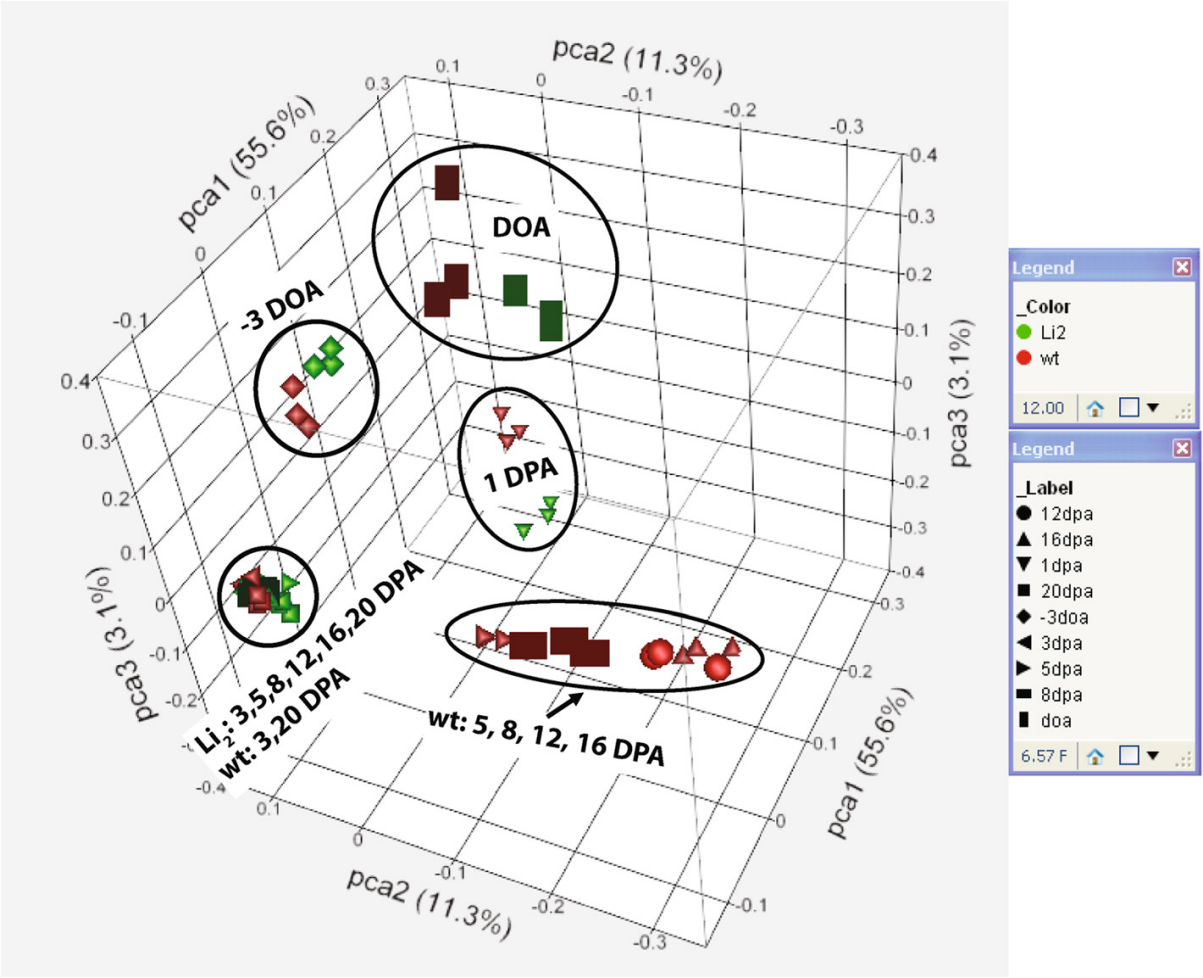
**Principal component analysis (PCA) of developing cotton fiber samples based on GC-MS metabolome from *Li***_***2 ***_**NILs. **Each time-point of developing cotton fiber is represented by 3 biological replicates. PCA was conducted using JMP Genomics 5 (SAS, Cary, NC, USA).

Nine time-points of fiber development representing overlapping stages of fiber initiation (−3 DOA, DOA, 1 DPA and 3 DPA), cell elongation (5, 8, 12 and 16 DPA) and beginning of secondary wall biosynthesis (20 DPA) were used for metabolite analysis. GC-MS analyses were performed for each sample of cotton ovules and fiber, and all peaks above the limit of detection, 487 in total, were subjected to statistical analysis. Additional file 2 provides normalized values and results of F test for each compound. Principal component analysis (PCA) was applied to explore relationship in metabolite pools among samples of *Li*_*2*_ mutant and WT NILs. Normalized peak areas of detected compounds were used as continuous variables. According to PCA, mutant and WT samples clustered together at early fiber developmental stages, separating −3 DOA, DOA, and 1 DPA time-points (Figure 2). During the elongation stage (5–16 DPA), WT samples were clearly separated from *Li*_*2*_ samples, indicating an alteration in the metabolome of the mutant fibers that corresponded with the loss of fiber elongation (Figure 2).

Of the 487 GC-MS detected peaks, 10 were not detected in *Li*_*2*_ mutant fiber samples. A two-way ANOVA was performed on the metabolome dataset with 215 peaks (44.1%) indicating a significant mutation effect (F ≤ 0.01), and 295 (60.6%) indicating a significant mutation x time-point interaction. Metabolites that demonstrated a significant mutation effect were further analyzed by two-dimensional hierarchical cluster analysis (HCA). The data for HCA analysis were prepared by dividing the mean value of peak area of each compound from 3 biological replicates of WT by the mean values of the mutant and then converting the mean ratios to log2 scale. HCA distributed metabolites into eight major clusters representing similar expression profiles. HCA also separated elongation stage of cotton fiber (5–16 DPA) into distinct clusters showing the closest distance in metabolite profiling between 12 and 16 DPA (Additional file 3A). Fifty three of the 487 GC-MS detected peaks were identified. Correlation analysis was performed between 51 identified metabolites to estimate interactions in the metabolomic network. The heatmap shown in Additional file 3B represents the correlations of selected compounds. Positive correlations (r^2^ > 0.65) were determined between free sugars along with amino acids, organic acids, sugar-alcohols, and phosphate intermediates, indicating cooperative regulation of distinctive metabolic pathways. Negative correlations were indicated by *p*-coumaric acid, 2-ketoglutaric acid, suberyl glycine, shikimic acid, serine, 5-hydroxytryptamine, and aspartic acid.

### Global transcript trends

Affymetrix microarray analyses were performed for samples at three developing time-points of cotton fiber, DOA, 8 DPA and 12 DPA, representing initiation and peak of elongation stages of development. Detailed descriptions of the microarray analyses were previously provided [10]. To interrogate possible biological processes affected by the *Li*_*2*_ mutation, parametric analysis of gene set enrichment (PAGE) was performed for microarray data using AGRIgo toolkit and database [http://bioinfo.cau.edu.cn/agriGO; [26]. PAGE employs fold change between parametric data to calculate Z scores of predefined gene sets and uses normal distribution to infer statistical significance of gene sets. Ratios (≥2) of differentially expressed probesets between *Li*_*2*_ and WT NILs at 8 DPA and 12 DPA were converted to Log2 values and submitted to PAGE analysis using Hochberg FDR adjusted p-value (≤0.05) cutoff and 10 entries as minimum mapping numbers. Among 4656 submitted probesets 3821 were annotated and assigned into 149 gene ontology (GO) terms, including 98 GO terms assigned to biological process, 18 GO terms assigned to molecular function and 33 GO terms assigned to cellular component. The list of annotated probesets and results of PAGE analysis are provided in Additional files 4 and 5.

The most strikingly up-regulated GO terms in *Li*_*2 *_mutant fiber were related to biological process such as component biogenesis and organization, which includes DNA conformation change, DNA packaging, chromosome organization, chromatin organization, protein-DNA complex assembly, and nucleosome assembly (Additional file 6A). Also, the list of significantly up-regulated GO terms included regulation of primary metabolic process, cellular macromolecule biosynthetic process, nitrogen compound metabolic process, glutamine family amino acid metabolic process, nucleic acid metabolic process, RNA and DNA metabolic processes, DNA replication, cell cycle process, response to stimulus and stress, and flavonoid metabolic process. Among down-regulated significantly enriched categories were lipid metabolism, signal transduction, intracellular transport, and polysaccharide catabolism. Analysis of GO terms in cellular component showed up-regulated genes associated with chromatin, nucleosome, nucleus, ribosome, and mitochondria, whereas genes related to cytoskeleton, cell wall, Golgi apparatus, and beta-galactosidase complex were down-regulated in *Li*_*2*_ fiber (Additional file 6B). Nucleic acid binding was the most enriched molecular function category up-regulated in *Li*_*2*_ fiber, whereas sugar binding, transferase activity and galactosidase activity were among down-regulated functional categories (Additional file 6C).

Thus, transcript analysis revealed that processes associated with DNA conformation change and replication were induced, whereas processes involved in polysaccharide biosynthesis, sugar transport, cell wall loosening and expansion were reduced in *Li*_*2*_ mutant fiber.

### Metabolism overview

**Figure 3:**
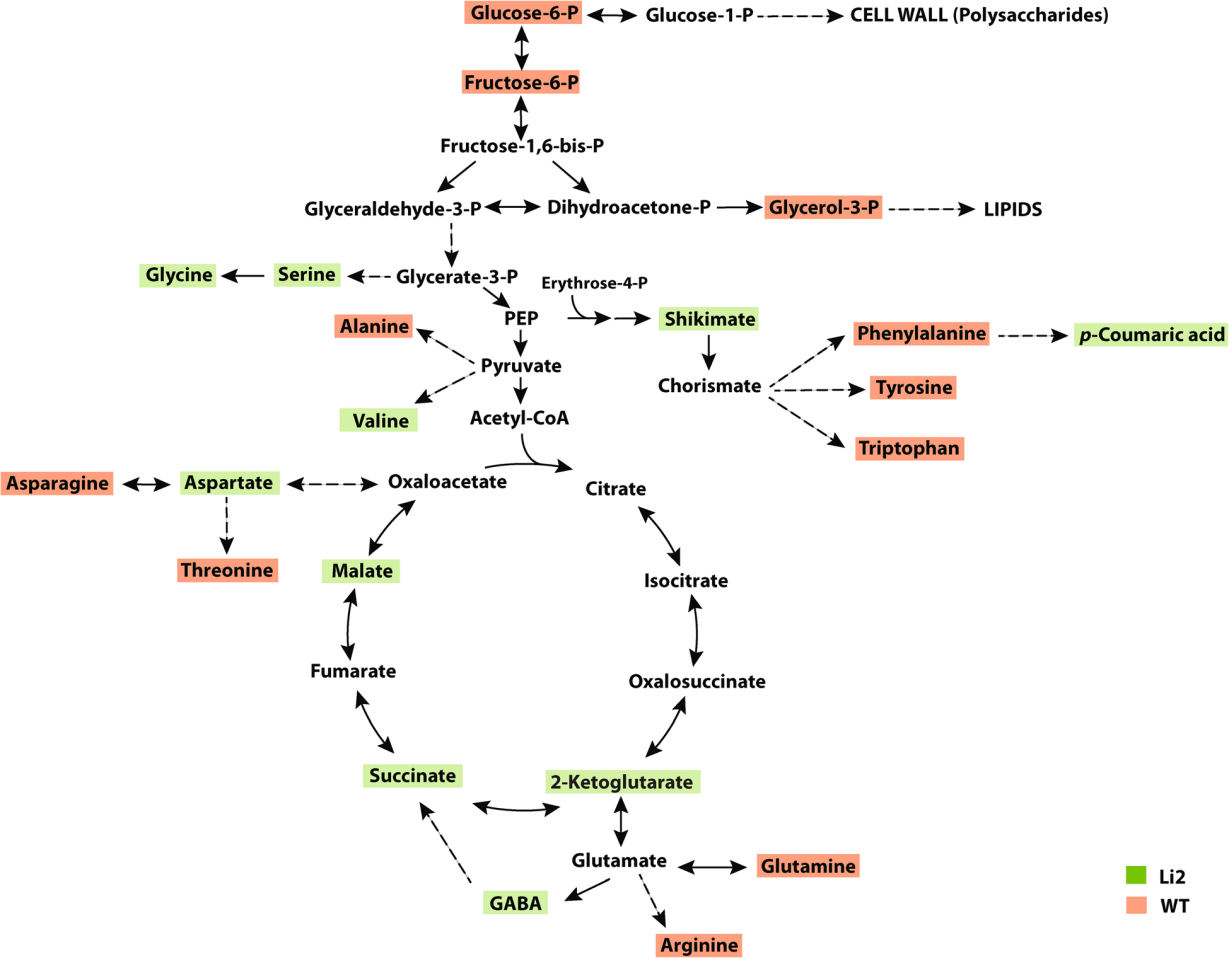
**Primary metabolism overview in elongating fibers of *Li***_***2 ***_**NILs. **Metabolites highlighted by red color are significantly up-regulated in WT, whereas metabolites highlighted by green color are significantly up-regulated in *Li*_*2*_ mutant elongating fiber in at least one time-point from 5 to 16 DPA (Table 1 and Table 2). Abbreviations: PEP, phospho*enol*pyruvate; GABA, gamma-aminobutyric acid.

**Figure 4:**
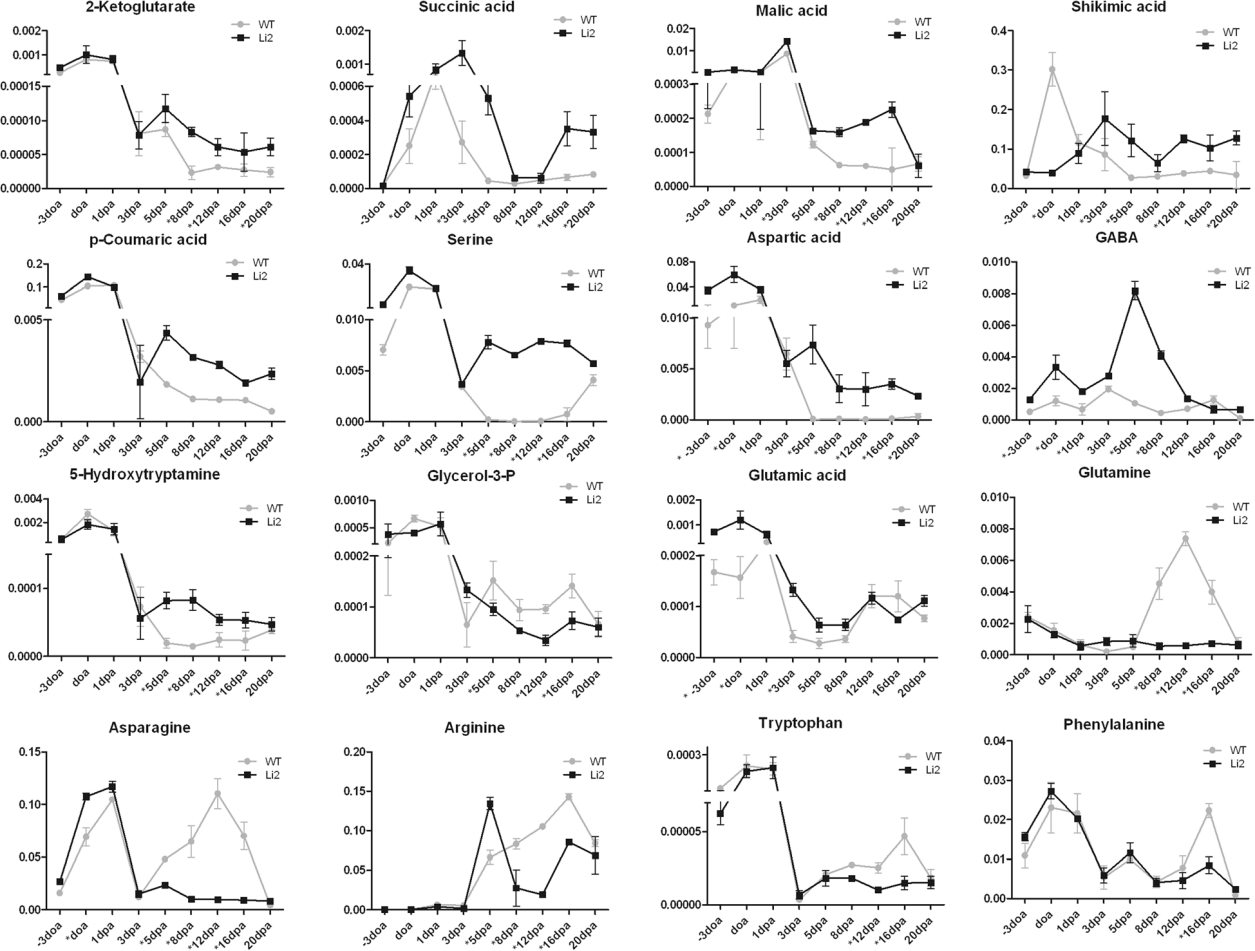
**Relative contents of selected metabolites in developing fibers of *Li***_***2 ***_**NILs. **Y-axis values represent relative peak areas after normalization to the mean peak area of the internal standards for a specific sample. Abbreviation: GABA, gamma-aminobutyric acid. Error bars represent standard deviation from three biological replicates. Asterisks indicate significant difference between lines.

Significant changes in the relative abundance of multiple identified metabolites were observed between *Li*_*2*_ NILs. Since overall metabolome analysis determined the major differences between NILs at elongation stage (from 5 DPA to 16 DPA) we focused our description for this stage of fiber development. To determine the differences in primary metabolism between *Li*_*2*_ NILs we schematically visualized changes in metabolites levels. As shown in Figure 3 metabolites highlighted by red color were significantly up-regulated in WT fibers, whereas metabolites highlighted by green color were significantly up-regulated in *Li*_*2*_ fibers in at least one time-point from 5 DPA to 16 DPA. Tables 1 and 2 represent fold changes in peak areas of metabolites up-regulated in elongating fibers of *Li*_*2*_ and WT plants. Organic acids, N-acetylglutamic acid, 2-ketoglutaric acid, malic acid, succinic acid, shikimic acid, and glycolic acid were significantly accumulated more in *Li*_*2*_ fibers, whereas oxalic acid, 2,3-dihydroxybutanedioic acid, maleic acid, ascorbic acid, 2-hydroxyglutaric acid, and isoascorbic acid were significantly higher in WT elongating fibers. The level of glycerol-3-phosphate, an important component of carbohydrate and lipid metabolic processes, was reduced in *Li*_*2*_ mutant fibers (Figures 3 and 4). Transcript data showed that carbohydrate and lipid metabolism were among down-regulated biological processes.

**Table 1:** **Fold change in peak area metabolites up-regulated in developing fiber of *Li***_***2 ***_**plants**

	**5DPA*Li***_***2***_**/WT**	**8DPA*Li***_***2***_**/WT**	**12DPA*Li***_***2***_**/WT**	**16DPA*Li***_***2***_**/WT**
Organic acids				
N-Acetylglutamic acid	**7.1**	**19.4**	**13.1**	**4.1**
2-Ketoglutaric acid	1.4	**3.6**	**1.9**	2.0
Succinic acid	**11.9**	2.3	1.3	**5.4**
Malic acid	1.3	**2.6**	**3.2**	**4.6**
Shikimic acid	**4.5**	2.1	**3.3**	**2.3**
Glycolic acid	**3.0**	**1.6**	1.3	1.1
Amino acids				
Serine	**36.2**	**328.8**	**142.8**	**10.5**
Aspartic acid	**126.5**	**36.5**	**63.2**	**31.2**
Valine	**5.0**	1.2	0.7	0.7
Glycine	**14.7**	1.1	0.5	2.4
Non-protein amino acid				
Gamma-Aminobutyric acid	**7.8**	**9.6**	**1.9**	0.5
Phenolic				
trans-p-Coumaric acid	**2.4**	**2.8**	**2.6**	**1.8**
Serotonin				
5-Hydroxytryptamine	**4.2**	**5.7**	**2.2**	2.3

Values represent fold change of mutant to WT in peak area of identified metabolites at 5, 8, 12, and 16 DPA. Bold font indicates significant changes (p < 0.05) in metabolite levels between near-isogenic lines (data represent 3 biological replicates).

**Table 2:** Fold change in peak area metabolites up-regulated in developing fiber of wild-type plants

	**5DPAWT/*Li***_***2***_	**8DPAWT/*Li***_***2***_	**12DPAWT/*Li***_***2***_	**16DPAWT/*Li***_***2***_
Organic acids				
Oxalic acid	**2.6**	0.9	1.6	**4.2**
2,3-Dihydroxybutanedioic acid	1.2	2.0	**2.8**	2.7
Maleic acid	0.2	**3.3**	1.2	**4.9**
Ascorbic acid	**4.2**	**7.7**	**11.0**	**21.2**
2-Hydroxyglutaric acid	**6.1**	**9.4**	**14.6**	**14.9**
Isoascorbic acid	**1.4**	**11.3**	**17.4**	**8.6**
Amino acids				
Phenylalanine	0.9	1.0	1.7	**2.6**
Tryptophan	1.1	**1.5**	**2.5**	**3.1**
Threonine	1.1	**5.0**	1.1	1.6
Alanine	2.0	**2.1**	**3.2**	**4.0**
Tyrosine	0.6	**3.1**	**2.0**	**6.3**
Asparagine	**2.1**	**6.4**	**11.2**	**7.6**
Glutamine	0.6	**8.3**	**12.6**	**5.5**
Arginine	**0.5**	**3.0**	**5.5**	**1.7**
Non-protein amino acid				
Pyroglutamic acid	**4.0**	1.2	**2.2**	**4.0**
Sugars, sugar alchohols, sugar acids, sugar phosphates				
Arabinose	**1.9**	**2.4**	**3.3**	**3.5**
Fructose	**6.5**	**13.4**	**27.5**	**66.6**
Glucose	**19.4**	**29.2**	**45.0**	**73.5**
Galactose	**7.4**	**21.8**	**46.8**	**78.3**
myo-Inositol	**0.6**	**1.6**	**4.0**	**4.7**
Trehalose	0.7	**1.5**	**1.6**	**2.1**
Maltose	0.9	**2.1**	**2.5**	**4.2**
Galactonic acid	1.6	**6.5**	**3.1**	**5.3**
Fructose-6-phosphate	**2.7**	**2.7**	**4.3**	**3.7**
Glucose-6-phosphate	1.9	**27.8**	**38.1**	**43.5**
Glycerol-3-phosphate				
Glycerol-3-phosphate	1.6	1.8	**2.8**	1.9

Values represent fold change of WT to mutant in peak area of identified metabolites at 5, 8, 12, and 16 DPA. Bold font indicates significant changes (p < 0.05) in metabolite levels between near-isogenic lines (data represent 3 biological replicates).

The amino acids serine and aspartic acid were significantly higher accumulated in *Li*_*2*_ mutant fibers during elongation (Figure 4). Valine and glycine showed higher levels in *Li*_*2*_ fibers only at 5 DPA. Phenylalanine, tryptophan, threonine, alanine, tyrosine, asparagine, glutamine, and arginine were significantly higher accumulated in WT fibers. Probesets related to genes involved in glutamine family amino acid metabolic processes, including two glutamine synthases [cytosolic Ghi.10275.1.S1_s_at [27] and chloroplastic Ghi.10775.1.S1_s_at], were highly induced in *Li*_*2*_ fibers (Additional file 7). Aspartate aminotransferase (AspAT) catalyzes the reversible transamination of oxaloacetate by glutamate to yield 2-ketoglutarate and aspartate. Two probesets of AspAT were higher induced in *Li*_*2*_ elongated fibers that may contribute to higher accumulation of 2-ketoglutarate and aspartate. Gamma-aminobutyric acid (GABA) is the decarboxylation product of glutamate. A Significantly higher level of GABA was observed in *Li*_*2*_ mutant fibers at 5 and 8 DPA.

Shikimic acid accumulated higher in *Li*_*2 *_fibers, whereas its downstream products, the aromatic amino acids phenylalanine, tyrosine, and tryptophan, were reduced. PAGE analysis determined significantly up-regulated GO terms involved in aromatic compound (phenylpropanoid and flavonoid) biosynthetic processes. Consistent with gene expression data, *p*-coumaric acid (a precursor for different branches of phenylpropanoid pathway) was higher in mutant fibers. 5-Hydroxytryptamine (serotonin), a product of tryptophan, was accumulated more significantly in *Li*_*2*_ fibers at the peak of elongation from 5 DPA to 16 DPA.

Genomic analysis by Shi *et al*. revealed that ethylene plays an important role during fiber elongation [28]. Three probesets (from 11 available on microarray) corresponding to 1-aminocyclopropane-1-carboxylic acid oxidases (ACOs) were higher induced in *Li*_*2*_ elongated fibers (Additional file 7). It has been shown that cytokinins stimulate ethylene production in etiolated seedling of Arabidopsis [29]. Elevated levels of cytokinins were also detected in ovules and the developing fibers of *Li* (authors did not specify if *Li*_*1*_ or *Li*_*2*_ was examined) mutant line [30]. High expression of ACO genes responsible for ethylene production in short mutant fiber may be induced by cytokinins.

### Sugars and cell wall polysaccharides biosynthesis

The highest content of non-cellulosic sugars was observed in ovules from −3 DOA up to 1 DPA, with no significant differences determined between fibers of the cotton NILs. Amounts of free sugars increased in WT fibers from 3 DPA up to 16 DPA and dropped at 20 DPA. All detected free sugars, sugar alcohols, sugar acids, and sugar phosphates were significantly reduced in *Li*_*2*_ fiber during elongation (Table 2).

**Figure 5:**
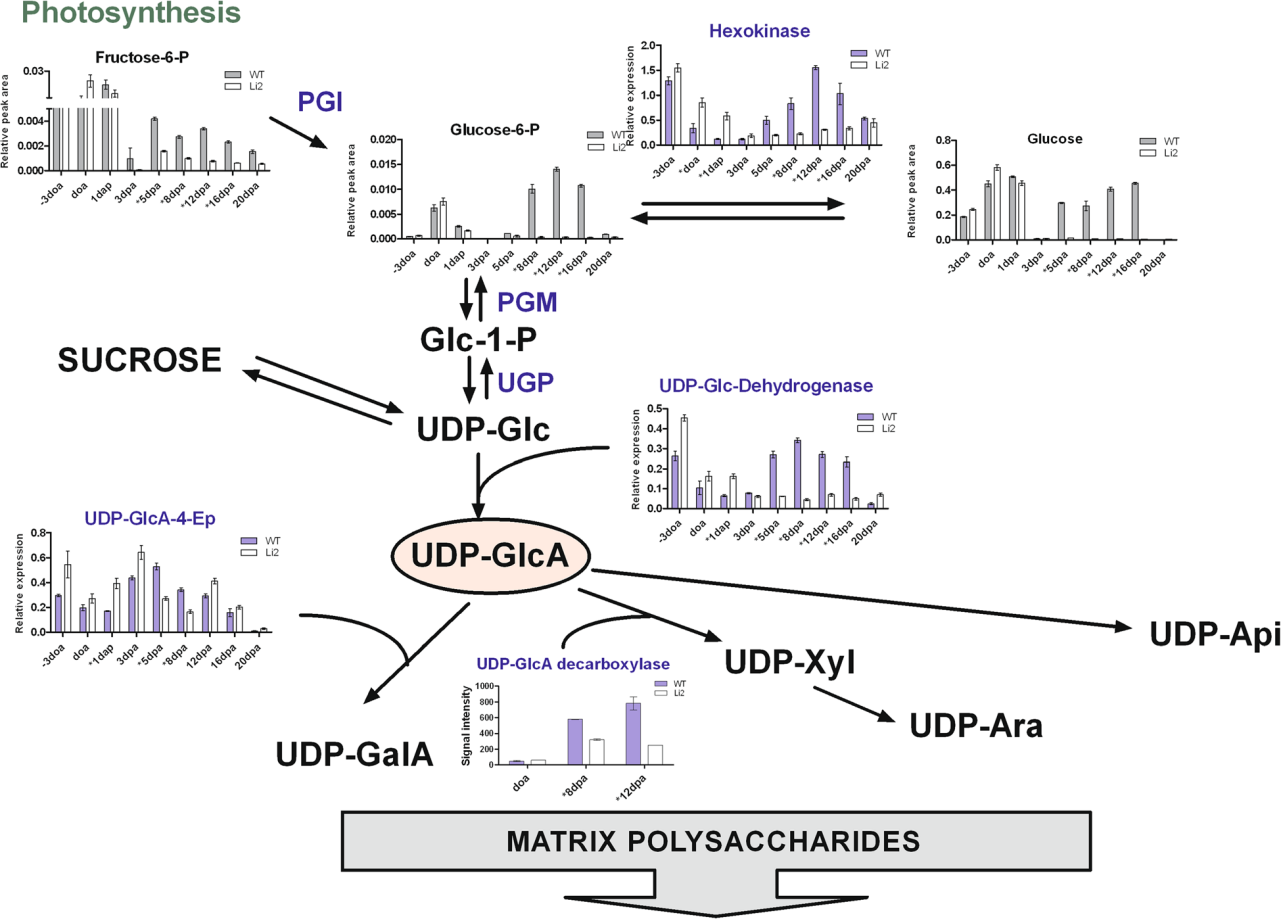
**Scheme of pathways for matrix polysaccharide precursors. **Bar charts represent relative metabolites levels and relative transcripts levels of enzymes involved in sugar-nucleotide interconversation in developing fibers between *Li*_*2 *_NILs. Error bars indicate standard deviation from three biological replicates. Asterisks represent significant difference between lines. Abbreviations: PGI, phosphoglucose isomerase; PGM, phosphoglucomutase; UGP, UDP-D-glucose pyrophosphorylase; UDP-GclA-4-Ep, UDP-glucuronic acid 4-epimerase; Fructose-6-P, fructose-6-phosphate; Glucose-6-P, glucose-6-phosphate; Glc-1-P, glucose-1-phosphate; UDP-Glc, UDP-glucose; UDP-GlcA, UDP-glucuronic acid; UDP-GalA, UDP-galacturonic acid; UDP-Xyl, UDP-xylose; UDP-Ara, UDP-arabinose; and UDP-Api, UDP-apiose.

The glycosyltransferases involved in carbohydrate biosynthesis typically depend on nucleotide sugars as substrates. Fructose-6-phosphate (Frc-6-P) is a major product of photosynthesis and a precursor for the formation of UDP-glucose (UDP-Glc) and other nucleotide sugars. The level of Frc-6-P was not significantly different at initiation stage between NILs, but was reduced 2–4 fold in *Li*_*2*_ fibers at elongation stage (Figure 5). Glucose-6-phosphate (Glc-6-P) provides sugar for different pathways of carbohydrate metabolism including matrix polysaccharide biosynthesis. The highest level of Glc-6-P was determined in elongating fibers of WT plants from 8 DPA to 16 DPA, whereas it was significantly reduced (up to background level) in *Li*_*2*_ fiber indicating a major role of Glc-6-P in biosynthesis of polysaccharides associated with cell wall extension. The functional characterization of an Arabidopsis plasma membrane-localized sugar transporter (*AtPLT5*) suggests that plants do have the ability to transport glycoses from the apoplast to the cytosol [31]. Microarray analysis determined that transcript levels of three monosaccharide transporters were significantly decreased at 8 DPA in mutant elongating fibers (Additional file 7). Hexokinase (HK) in plants phosphorylates several hexoses including glucose, fructose, mannose, and galactose [32]. Transcript levels of two HKs detected by microarray were significantly down-regulated in *Li*_*2*_ elongating fibers. RT-qPCR analysis of HK (probeset Ghi.7480.1.A1_at) confirmed significant transcript reduction in *Li*_*2*_ fibers during elongation whereas this gene was up-regulated in mutant line during initiation (Figure 5). Sucrose synthase (SuSy) may also contribute to the UDP-Glc pool by reversible conversion of sucrose and UDP into UDP-Glc and fructose. However, the transcript profile of previously characterized *SuSy3*[33,34] did not show significant differences between NILs during fiber elongation [10].

UDP-glucuronic acid (UDP-GlcA) is the central intermediate in the interconversion pathway to other nucleotide sugars, including the UDP-derivatives of xylose, arabinose, apiose, and galacturonic acid, which are precursors for matrix polysaccharides formation [35]. UDP-glucose dehydrogenase (UGD) converts UDP-Glc to UDP-GlcA. UGD (probeset Ghi.8627.1.S1_s_at) has 90% amino acid identity to *UGD1* (NCBI accession ACJ11712) and showed significant transcript reduction during elongation stage in *Li*_*2*_ mutant fibers (Figure 5). Proteomic study of cotton developing fiber determined association of *UGD1* with fiber elongation [36]. Pairwise correlation analysis revealed 73% correlation (p-value 0.0006) in transcript profiles between HK and UGD, indicating cooperative involvement of these genes in matrix polysaccharide biosynthesis during fiber elongation. Young cotton fibers have a bilayered primary cell wall consisting of an inner layer rich in cellulose and xyloglucans and an outer sheath rich in pectin [37]. UDP-galacturonic acid (UDP-GalA) is a major sugar residue of plant pectic polysaccharides, whereas UDP-Xylose (UDP-Xyl) is a main component of xyloglucan in primary cell walls [38]. UDP-glucuronic acid 4-epimerase (GAE) catalyzes the epimerization of UDP-GlcA to UDP-GalA. The Affymetrix cotton microarray contains a probeset (Gra.2659.2.S1_s_at) corresponding to previously characterized *GAE3* (GB ADB24770), which was highly preferentially expressed in fast elongating fiber cells [36]. Microarray did not determine significant changes in transcript level of *GAE3* in fibers of the NILs, however, more accurate RT-qPCR analysis revealed down-regulation two fold in *Li*_*2*_ fibers (Figure 5). Transcript level of a gene encoding an enzyme involved in formation of UDP-Xyl, UDP-glucuronic acid decarboxylase (GAD), was down-regulated 2–3 fold in *Li*_*2*_ fiber at 8 DPA and 12 DPA.

**Figure 6:**
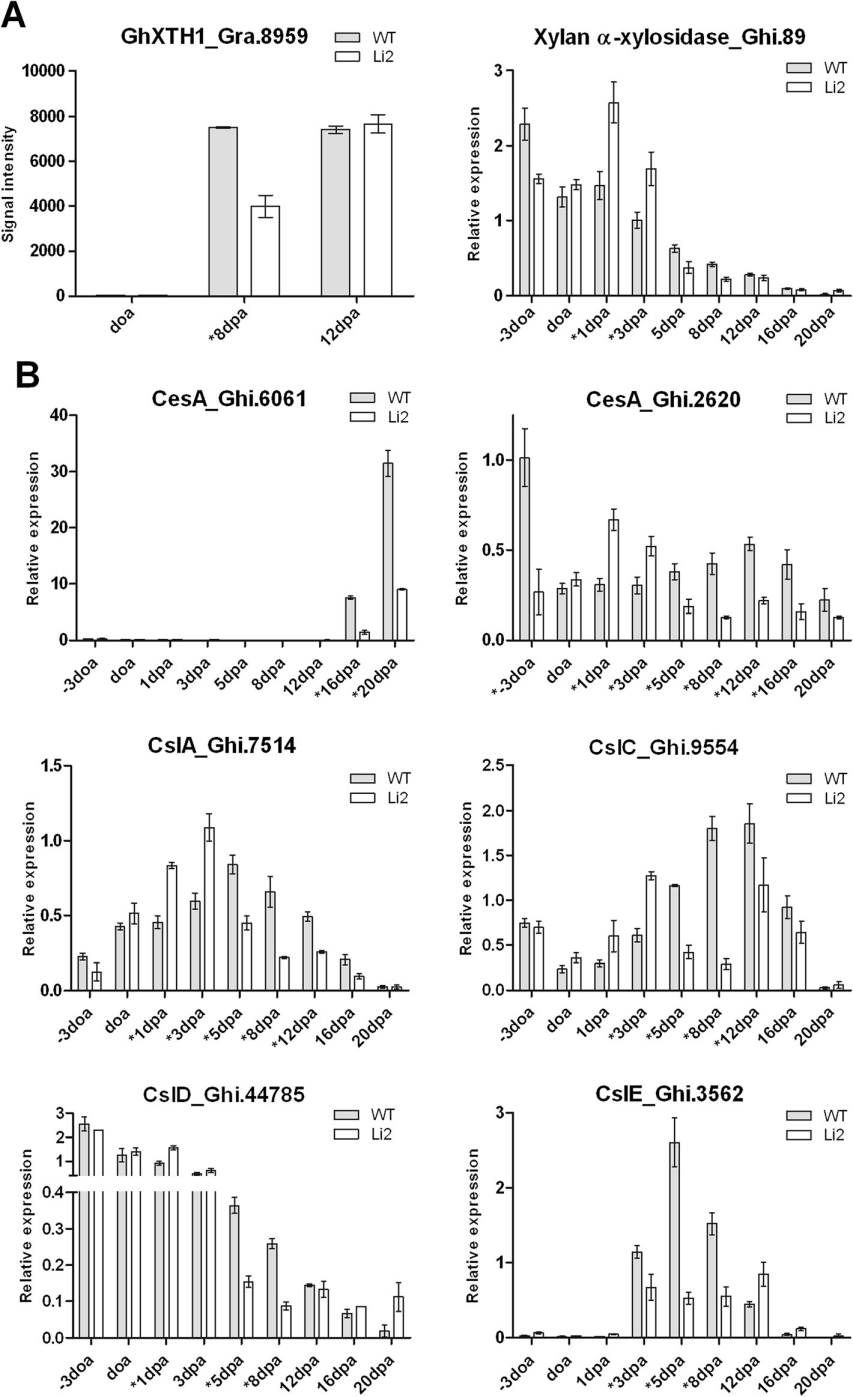
**RT-qPCR analysis of genes involved in hydrolysis and biosynthesis of polysaccharides during fiber elongation in *Li***_***2 ***_**NILs. **Probeset identifications and primers for genes encoding (**A**) glycoside hydrolyzing enzymes; and (**B**) cellulose synthase like superfamily members provided in Additional file 1. Error bars indicate standard deviation from three biological replicates. Asterisks represent significant difference between lines.

Hydrolysis of xyloglucans and pectins has been associated with regulation of cotton fiber elongation [39]. It was previously shown the involvement of β-galactosidases in pectin degradation in cell walls [40]. PAGE analysis determined that β-galactosidase gene family was one of the most noticeably down-regulated GO terms with 13 probe sets corresponding to β-galactosidases significantly down-regulated in *Li*_*2*_ mutant fibers. In our previous report, RT-qPCR analysis of β-galactosidase [Gra.2056.1.A1_s_at; [41]] that was preferentially expressed in cotton fiber showed no expression in *Li*_*2*_ mutant fiber [10]. This suggests that hydrolysis of pectin polymers is reduced in *Li*_*2*_ mutant fibers during elongation. Genes encoding xyloglucan modifying enzymes were also tested and revealed 8 probe sets of xyloglucan endotransglycosylases (XTHs) were down-regulated in *Li*_*2*_ fibers (Additional file 7). The previously characterized *GhXTH1*[42], that when overexpressed in cotton resulted in up to a 20% increase in fiber length, was down-regulated 2 fold at 8 DPA in mutant fibers (Figure 6A). However, the expression of xylan α-xylosidases that cleave 1,6-α-xylosyl residues from 1,4-β-glucan backbone did not differ significantly between fibers of the NILs or were up-regulated in *Li*_*2*_ fibers suggesting that xylosyl side chain removal is not important for elongation.

Multiple probe sets corresponding to genes of cellulose synthase (CesA) and cellulose synthase like (CSL) families were down-regulated in *Li*_*2*_ mutant fibers. We evaluated expression of CesA and CSL family members by RT-qPCR (Figure 6B). Members of the cellulose synthase A (CesA) gene family showed two different expression profiles demonstrating involvement into secondary cell wall (Ghi.6061) and primary cell wall (Ghi.2620) biosynthesis with transcripts corresponding to both probe sets down-regulated in *Li*_*2*_ mutant fibers. CslA, CslC, and CslE genes were highly induced during elongation (from 3 DPA to 16 DPA) in WT fibers, whereas transcripts were significantly reduced in *Li*_*2*_ mutant fibers. Peak expression level of CslD gene was detected at initiation stage and reduced during elongation. The expression level of CslD was also two fold down-regulated in *Li*_*2*_ fibers compared to WT fibers at 5 DPA - 8 DPA.

## Discussion

**Figure 7:**
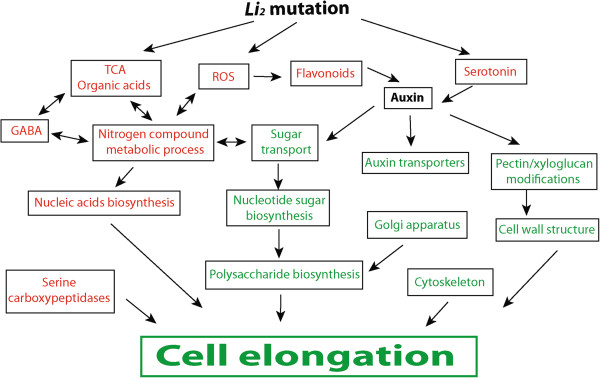
**Proposed model of the down-stream synergistic effects of the *Li***_***2 ***_**mutation on biological processes during fiber cell elongation. **Metabolites and GO terms associated with biological processes induced (red font) or reduced (green font) in mutant fibers. *Li*_*2 *_mutation initiates genetic reprogramming of primary metabolism that results in reduction of cell elongation. TCA organic acids are higher accumulated in *Li*_*2 *_fiber indicating higher nitrate assimilation. Levels of nitrogen transport amino acids with coordinated expression of genes involved in nitrogen compound metabolic processes suggested redirection of carbon flow into nitrogen metabolism in mutant fiber. GABA might be involved in regulation of nitrogen metabolism and consequently in cotton fiber elongation. Interactions between ROS and cell cycle can be related to fiber elongation process. Down regulation of biological processes involved in cell wall expansion such as carbohydrate biosynthesis, cell wall loosening and cytoskeleton results in shorter cotton fiber.

Cotton fiber cell development is a complex and highly regulated process involving many metabolic pathways, signal transduction, and transcriptional regulation machineries. The *Li*_*2*_ cotton mutant line with extremely shortened lint fibers provides an excellent system to study cotton fiber elongation. In this article we report a comprehensive analysis of changes caused by *Li*_*2*_ mutation during cotton fiber development using GC-MS based metabolite profiling and transcriptomics approaches. It was previously shown that cotton fiber developmental stages can be separated by both gene expression and metabolite profiles [43]. Three overlapping stages of fiber initiation, cell elongation, and beginning of secondary wall deposition were evaluated by GC-MS analysis. PCA of GC-MS data determined that the *Li*_*2*_ mutation altered the metabolome of the mutant fibers compared to WT fibers. An overview of metabolic pathways and biological processes altered by *Li*_*2*_ mutation is illustrated in Figure 7 and discussed below.

The incorporation of nitrogen into organic compounds represents a rate-limiting step in biomass production and may indirectly control the elongation process. There is a complex interaction between nitrogen and carbon metabolism as nitrate leads to an orchestrated change in gene expression, which facilitates a reprogramming of carbon metabolism [44]. PAGE analysis determined that nitrogen compound metabolism, including biosynthesis glutamine family amino acids and nucleic acids, were significantly up-regulated GO terms in *Li*_*2*_ mutant fibers. It has been shown that ethylene induces glutamine synthetase activity in *Hevea brasiliensis* latex cells [45]. Higher expression of ethylene biosynthetic genes correlates with higher transcript activity of glutamine synthetase in *Li*_*2*_ mutant fibers. Levels of nitrogen transport amino acids, glutamate, glutamine, aspartate, and asparagine, were significantly perturbed in *Li*_*2*_ elongating fibers. Glutamine is a critical donor for nucleotides synthesis and required as substrate for three enzymes involved in the *de novo* synthesis of purine nucleotides and two enzymes involved in the *de novo* synthesis of pyrimidine nucleotides [46]. The low level of detected glutamine, despite high induction of genes encoding glutamine biosynthetic enzymes, can be explained by its higher demand for the nucleotides biosynthesis in *Li*_*2*_ elongating fibers.

It is known that nitrate assimilation is closely integrated with changes in organic carbon metabolism. During nitrate assimilation carbohydrate synthesis is decreased and more carbon entered into organic acid metabolism [44]. Therefore higher accumulation of organic acids (2-ketoglutarate, succinate, and malate) in *Li*_*2*_ mutant fibers suggests higher nitrate assimilation. Organic acids have two distinct functions during nitrate assimilation: malate acts as a counter-anion and prevents alkalinization during nitrate assimilation [47]; and 2-ketoglutarate is the primary carbon acceptor for ammonium in the GOGAT pathway [44]. Plants can sense their nitrogen status and regulate the uptake and reduction of nitrate adequately. Significantly higher accumulation of GABA was determined in *Li*_*2*_ mutant fiber. GABA is a non-protein amino acid that accumulates rapidly in plant tissues under a variety of stresses and is known as a signaling factor in many organisms. There have been a number of suggestions regarding the role of GABA in regulation of nitrogen metabolism. GABA affects growth and development of Arabidopsis seedlings differently depending on the concentration of inorganic nitrogen in the growth medium. Root growth was stimulated by the addition of GABA at low nitrate concentrations, whereas high nitrate concentrations inhibited root elongation [48]. A signaling role of GABA was suggested due to the disruptive effect of GABA metabolism on plant development. Transgenic plants overexpressing glutamate decarboxylase have significantly reduced levels of glutamate with extremely high GABA and exhibit severe morphological abnormalities, such as short stems, in which cortex parenchyma cells fail to elongate [49]. Further evidence for a signaling role of GABA in plants comes from analysis of Arabidopsis *pop2* mutants, which are impaired in the first step of GABA degradation. Flowers of *pop2* mutants with low levels of GABA (200 uM) displayed normal guidance, while increases in GABA to 100-fold above that level caused severe defects in guidance *in vivo* and decreased pollen tube growth *in vitro*[50]. A more recent study determined an additional growth inhibition effect of exogenous GABA application on primary root and dark-grown hypocotyls in *pop2* mutants due to cell elongation defects [51]. Notably, in all examples, alterations in GABA levels were found to have an effect on cell elongation. The higher accumulation of GABA in *Li*_*2*_ mutant fibers suggests involvement of GABA in fiber elongation process. It is still unclear which factor initiated inhibition of elongation in *Li*_*2*_ fibers and how GABA is related to this process. Consequently, further investigation is required to clearly elucidate the role of GABA in cotton fiber elongation.

Transcriptional activation of GO terms related to DNA conformation change, DNA biosynthesis and cell cycle in mutant fibers suggests that S-phase replication process is more active in *Li*_*2*_ fibers. However, our evaluation of field grown *Li*_*2*_ fibers at different developmental stages did not reveal any visible multicellular fibers. Multicellular fibers are rare events and have been reported only in cotton ovule cultures grown in the absence of exogenous hormones. Adding the indole-3-acetic acid (IAA) to the medium stimulate fibers to expand as single cells [52]. We did not determine significant changes in transcript levels of genes involved in IAA biosynthesis between NILs, however, auxin transporters were significantly down-regulated in *Li*_*2*_ fibers (Additional file 7). Higher accumulation of serotonin observed in mutant fibers is interesting since serotonin acts as a natural auxin inhibitor in plants. More specifically, high doses of serotonin repressed lateral root growth, primary root growth, and root hair development in Arabidopsis [53]. Therefore, serotonin may be one of the factors indirectly repressing the elongation process in *Li*_*2*_ mutant fibers.

Several other observations from our analyses were also interesting. A higher activation of genes involved in stress-response processes suggests elevated levels of reactive oxygen species (ROS) in *Li*_*2*_ mutant fibers. ROS can be generated during diverse biological and cellular reactions and act either positively for biological activity or negatively resulting in toxicity. The involvement of ROS for cell wall extension is well documented [54-57], although regulation of ROS homeostasis is necessary for proper cell elongation [58,59]. In our previous report, based on transcriptional regulation of the list of genes involved in the ROS production and repair of oxidative damage, we concluded that ROS homeostasis is compromised in *Li*_*2*_ mutant fibers [10]. Additionally there is a massive induction of phenylpropanoid genes in *Li*_*2*_ mutant fibers and accumulation of *p*-coumaric acid, which is an early precursor for different branches in phenylpropanoid pathway (Additional file 7, Figure 4). There are a number of reports observing higher induction of flavonoid genes in shorter fiber cotton species [59-61]. Previous studies demonstrated that flavonoids accumulation act as negative regulators of auxin transport [62-66]. Indeed, transcriptional activity of auxin transporters was reduced significantly in *Li*_*2*_ mutant fibers. However, flavonoids are not specific regulators of auxin transporters since the major role of flavonoids is on auxin or auxin oxidative products themselves [67]. A primary role of flavonoids is scavenging ROS, which suggests the activation of phenylpropanoid genes in *Li*_*2*_ mutant fibers is in response to elevated levels of ROS.

Recent studies also indicated that ROS play a critical role in signal transduction and cell cycle regulation [68]. It was noted that in synchronized cells the production of ROS increases during the cell cycle, with peak levels occurring in the G2/M phase [69,70]. The regulation of cell cycle itself is unusual in cotton fibers [71] and DNA content relative to cell cycle status has been explored in developing *Gossypium hirsutum* fibers [71,72]. A 25% increase in DNA content in 3 and 5 DPA fibers relative to DOA fibers was observed at entry into the S-phase of the cell cycle [72]. In another study, it was determined that no endoreduplication occurred and there was no increase in genome size, although there was an apparent increase in S-phase cells as fibers developed and matured. Also, the low level of expression of genes associated with cell cycle progression suggested that S-phase arrest occurs in developing cotton fibers [71]. In our study we observed in *Li*_*2*_ mutant fibers higher transcript activities of genes related to replication as well as induction of ROS responding genes. There are two conflicting possibilities regarding interactions between ROS and cell cycle in *Li*_*2*_ mutant elongating fibers: (1) genes related to S-phase replication are activated in response to ROS or, (2) the level of ROS increased due to compromised cell cycle. Although without careful evaluation of DNA content in *Li*_*2*_ mutant fibers it would be too early to make conclusions.

Strict down-regulation of GO terms associated with cell wall extension biological processes and cellular components were observed in *Li*_*2*_ mutant fibers. Transcriptional activity of genes related to carbohydrate metabolism was reduced in mutant fibers and coincided with a significant reduction of all detected sugars. It was previously demonstrated that nucleotide sugar metabolism plays a central role in cotton fiber elongation. Comparative proteomics studies of developing fibers of WT compared to fuzzless-lintless mutant plants revealed that nucleotide sugar metabolism was the most significantly up-regulated biochemical process during fiber elongation [36]. Glc-6-P along with multiple genes encoding sugar interconversion enzymes and sugar transporters were significantly down-regulated suggesting that biosynthesis of sugar nucleotides is reduced in developing *Li*_*2*_ mutant fiber cells. GO terms associated with cellular components, such as cytoskeleton and Golgi apparatus, were among down-regulated biological processes in *Li*_*2*_ mutant fibers. The importance of actin cytoskeleton in fiber elongation was reported earlier. Down-regulation of *GhACT1* in cotton disrupted the actin cytoskeleton network in fibers that resulted in inhibition of fiber elongation [73]. Biosynthesis of non-cellulosic polysaccharides is known to take place in Golgi organelle [74-76]. The matrix polysaccharides are dominant constituents in cell walls of growing cotton fibers [77]. The active biosynthesis of matrix polysaccharides along with increased activity of cell wall loosening enzymes has been considered to be associated with cell wall extension [39,77-80]. Our evaluation of transcript levels of genes encoding polysaccharide biosynthesis and cell wall loosening enzymes determined significant transcript reduction for both classes of enzymes in *Li*_*2*_ mutant elongating fibers. Therefore, biosynthesis and modifications of matrix polysaccharides are reduced in mutant fiber.

## Conclusion

This report provides the first comprehensive analysis of metabolite and transcript changes in response to *Li*_*2*_ mutation in elongating fibers. PCA of GC-MS data separated mutant fiber samples from WT fiber samples at the elongation stage, indicated alterations in the metabolome of the mutant line as a result of the *Li*_*2*_ mutation. Increased amounts of TCA organic acids and induction of GO terms related to nitrogen compound metabolic processes, concurrent with the down regulation of carbohydrate metabolism, suggested redirection of carbon flow into nitrogen metabolism in *Li*_*2*_ mutant fibers. A number of factors associated with cell elongation found in this study will facilitate further research in understanding metabolic processes of cotton fiber elongation.

## Abbreviations

DOA: Day of anthesis; DPA: Days post-anthesis; GABA: Gamma-aminobutyric acid; GO: Gene ontology; NIL: Near-isogenic line; ROS: Reactive oxygen species; RT-qPCR: Reverse transcription quantitative polymerase chain reaction.

## Competing interests

The authors declare that they have no competing interests.

## Authors’ contributions

MN had the main responsibility for this research; performed sample preparation, extraction and GC/MS metabolite analysis; wrote the manuscript. DJH was responsible for the greenhouse experiment; tagging and sample harvest; RNA isolations; RT-qPCR and microarray analysis. RBT developed the Li2 mutant and WT NILs. JMB run GC/MS and was involved in the data analysis. DDF conceived the project. All authors read and approved the final manuscript.

## Supplementary Material

Additional file 1 **Primers used in RT-qPCR experiment. **The forward and reverse primer’s sequences and their PCR efficiencies are shown. Click here for file

Additional file 2 Normalized GC-MS metabolite profiling data and results of F test for each compound. Click here for file

Additional file 3 **Metabolic changes associated with *Li***_***2 ***_**mutation. **(A) Hierarchical clustering analysis of metabolite data of developing cotton fiber samples from *Li*_*2 *_near-isogenic lines. Metabolites correlated with *Li*_*2 *_mutation effects were analyzed by two-dimensional hierarchical cluster analysis using ward method. (B) Visualization of identified metabolite correlations. Heatmap of correlations (ratios WT versus *Li*_*2 *_converted to log2 scale) along cotton fiber developmental time-points from −3 DOA to 20 DPA. Correlation coefficients were calculated by applying Pairwise method using JMP genomics 5 software. Each square indicates a given r value resulting from a Pairwise correlation analysis in a false color scale. Click here for file

Additional file 4 The list of annotated probesets used for parametric analysis of gene set enrichment. Click here for file

Additional file 5 The result of parametric analysis of gene set enrichment. Click here for file

Additional file 6 **Parametric analysis of gene set enrichment of differentially expressed genes in elongating fibers of *Li***_***2 ***_**NILs at 8 DPA (col1) and 12 DPA (col2). **Graphical result of GO terms related to (A) biological process, (B) cellular component and (C) molecular function. Red indicates up-regulated terms; blue indicates down-regulated terms; and numbers indicate the adjusted p-value of the term at each time-point. Click here for file

Additional file 7 **Selected probesets representing gene families differentially expressed at 8 DPA or 12 DPA between *Li***_***2 ***_**NILs.** Click here for file
