# Supplementary figures and images for: Integrated metabolomics and genomics analysis provides new insights into the fiber elongation process in Ligon lintless-2 mutant cotton (Gossypium hirsutum L.)
Source: BMC Genomics. 2013 Mar 7;14:155. doi: 10.1186/1471-2164-14-155 (PMC3605188; doi:10.1186/1471-2164-14-155)

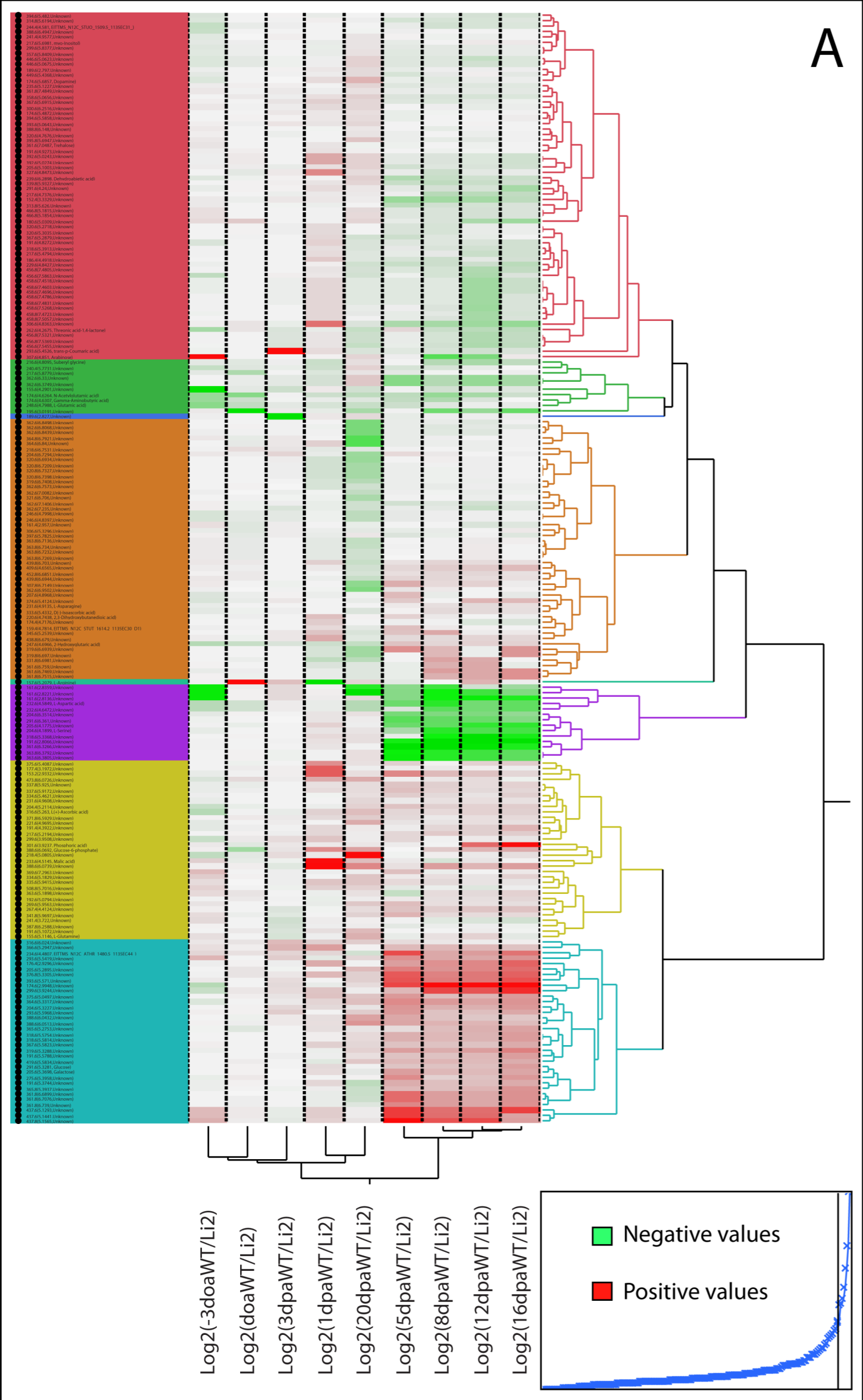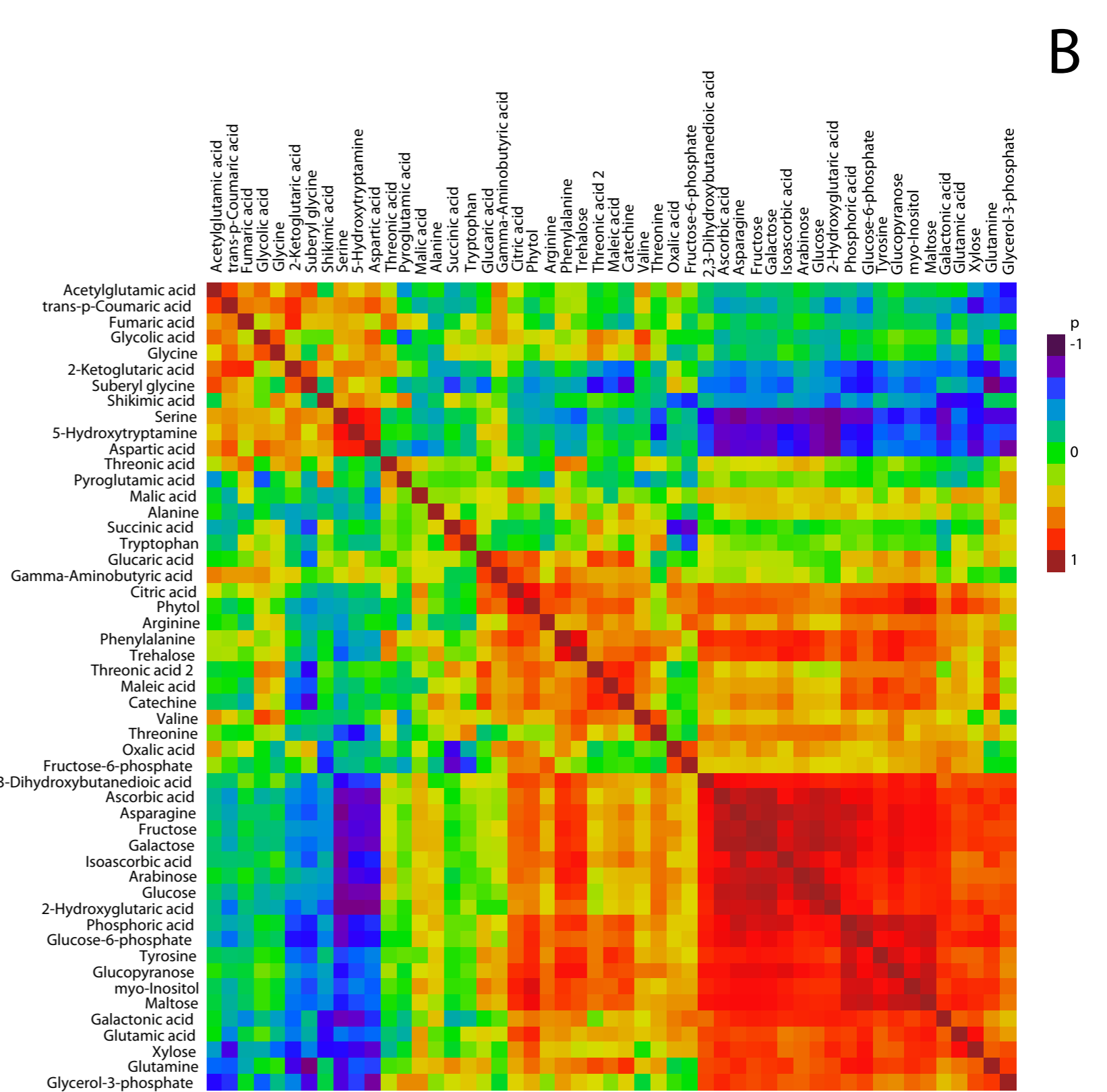

Supplement: Additional file 3 — Metabolic changes associated with Li2 mutation. (A) Hierarchical clustering analysis of metabolite data of developing cotton fiber samples from Li2 near-isogenic lines. Metabolites correlated with Li2 mutation effects were analyzed by two-dimensional hierarchical cluster analysis using ward method. (B) Visualization of identified metabolite correlations. Heatmap of correlations (ratios WT versus Li2 converted to log2 scale) along cotton fiber developmental time-points from −3 DOA to 20 DPA. Correlation coefficients were calculated by applying Pairwise method using JMP genomics 5 software. Each square indicates a given r value resulting from a Pairwise correlation analysis in a false color scale. [file 1471-2164-14-155-S3.pdf]
