# Supplementary material for: Integrated metabolomics and genomics analysis provides new insights into the fiber elongation process in Ligon lintless-2 mutant cotton (Gossypium hirsutum L.)
Source: BMC Genomics. 2013 Mar 7;14:155. doi: 10.1186/1471-2164-14-155 (PMC3605188; doi:10.1186/1471-2164-14-155)
Supplement: Additional file 6 — Parametric analysis of gene set enrichment of differentially expressed genes in elongating fibers of Li2 NILs at 8 DPA (col1) and 12 DPA (col2). Graphical result of GO terms related to (A) biological process, (B) cellular component and (C) molecular function. Red indicates up-regulated terms; blue indicates down-regulated terms; and numbers indicate the adjusted p-value of the term at each time-point. [file 1471-2164-14-155-S6.pdf]

A

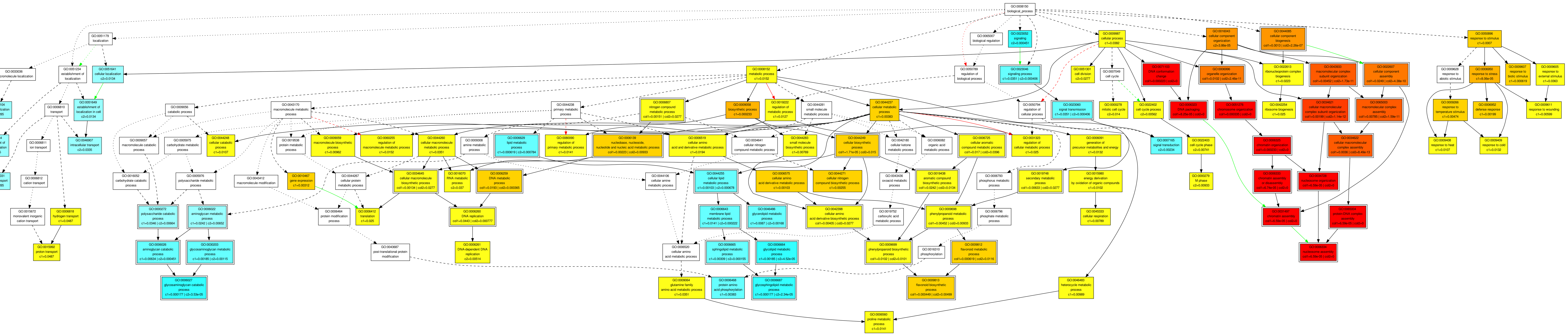

GO:0008150 biological_process
GO:0051179 localization
GO:0033036 macromolecule localization
GO:0051234 establishment of localization
GO:0051641 cellular localization c2=0.0134
GO:0008104 protein localization c2=0.0285
GO:0006810 transport
GO:0051649 establishment of localization in cell c2=0.0134
GO:0045184 establishment of protein localization c2=0.0285
GO:0006811 ion transport
GO:0046907 intracellular transport c2=0.0335
GO:0015031 protein transport c2=0.0285
GO:0006812 cation transport
GO:0015672 monovalent inorganic cation transport
GO:0006818 hydrogen transport c1=0.0487
GO:0015992 proton transport c1=0.0487
GO:0009056 catabolic process
GO:0043170 macromolecule metabolic process
GO:0009057 macromolecule catabolic process
GO:0005975 carbohydrate metabolic process
GO:0044248 cellular catabolic process c1=0.0107
GO:0019538 protein metabolic process
GO:0009059 macromolecule biosynthetic process c1=0.00962
GO:0060255 regulation of macromolecule metabolic process c1=0.0152
GO:0016052 carbohydrate catabolic process
GO:0005976 polysaccharide metabolic process
GO:0043412 macromolecule modification
GO:0010467 gene expression c1=0.00312
GO:0044267 cellular protein metabolic process
GO:0000272 polysaccharide catabolic process c1=0.0346 | c2=0.00664
GO:0006022 aminoglycan metabolic process c1=0.0242 | c2=0.00652
GO:0006464 protein modification process
GO:0006412 translation c1=0.025
GO:0006026 aminoglycan catabolic process c1=0.00634 | c2=0.000451
GO:0030203 glycosaminoglycan metabolic process c1=0.00185 | c2=0.00115
GO:0043687 post-translational protein modification
GO:0006027 glycosaminoglycan catabolic process c1=0.000177 | c2=3.53e-05
GO:0008152 metabolic process c1=0.0152
GO:0044238 primary metabolic process
GO:0006807 nitrogen compound metabolic process col1=0.00151 | col2=0.0277
GO:0009058 biosynthetic process c1=0.000233
GO:0019222 regulation of metabolic process c1=0.0127
GO:0044281 small molecule metabolic process
GO:0044237 cellular metabolic process c1=0.00383
GO:0044260 cellular macromolecule metabolic process c1=0.0351
GO:0009308 amine metabolic process
GO:0006629 lipid metabolic process c1=0.000619 | c2=0.000784
GO:0080090 regulation of primary metabolic process c1=0.0141
GO:0006139 nucleobase, nucleoside, nucleotide and nucleic acid metabolic process col1=0.00223 | col2=0.00933
GO:0006519 cellular amino acid and derivative metabolic process c1=0.0194
GO:0034641 cellular nitrogen compound metabolic process
GO:0044283 small molecule biosynthetic process c1=0.00769
GO:0044249 cellular biosynthetic process col1=1.71e-05 | col2=0.015
GO:0042180 cellular ketone metabolic process
GO:0006082 organic acid metabolic process
GO:0006725 cellular aromatic compound metabolic process col1=0.017 | col2=0.0396
GO:0031323 regulation of cellular metabolic process c1=0.025
GO:0006091 generation of precursor metabolites and energy c1=0.0132
GO:0034645 cellular macromolecule biosynthetic process col1=0.00134 | col2=0.0277
GO:0016070 RNA metabolic process c2=0.037
GO:0006259 DNA metabolic process col1=0.0193 | col2=0.000365
GO:0044106 cellular amine metabolic process
GO:0044255 cellular lipid metabolic process c1=0.00103 | c2=0.000678
GO:0006575 cellular amino acid derivative metabolic process c1=0.00103
GO:0044271 cellular nitrogen compound biosynthetic process c1=0.00255
GO:0043436 oxoacid metabolic process
GO:0019438 aromatic compound biosynthetic process col1=0.0242 | col2=0.0134
GO:0006793 phosphorus metabolic process
GO:0019748 secondary metabolic process col1=0.00633 | col2=0.0277
GO:0015980 energy derivation by oxidation of organic compounds c1=0.0102
GO:0006260 DNA replication col1=0.0443 | col2=0.000777
GO:0006643 membrane lipid metabolic process c1=0.0141 | c2=0.000222
GO:0046486 glycerolipid metabolic process c1=0.0087 | c2=0.00168
GO:0042398 cellular amino acid derivative biosynthetic process col1=0.00405 | col2=0.0277
GO:0019752 carboxylic acid metabolic process
GO:0009698 phenylpropanoid metabolic process col1=0.00452 | col2=0.00933
GO:0006796 phosphate metabolic process
GO:0045333 cellular respiration c1=0.00789
GO:0006261 DNA-dependent DNA replication c2=0.00514
GO:0006520 cellular amino acid metabolic process
GO:0006665 sphingolipid metabolic process c1=0.00309 | c2=0.000155
GO:0006664 glycolipid metabolic process c1=0.00185 | c2=4.52e-05
GO:0009699 phenylpropanoid biosynthetic process col1=0.0102 | col2=0.0101
GO:0016310 phosphorylation
GO:0009812 flavonoid metabolic process col1=0.000619 | col2=0.0116
GO:0009064 glutamine family amino acid metabolic process c1=0.0351
GO:0006468 protein amino acid phosphorylation c1=0.00383
GO:0006687 glycosphingolipid metabolic process c1=0.000177 | c2=2.34e-05
GO:0009813 flavonoid biosynthetic process col1=0.000449 | col2=0.00499
GO:0046483 heterocycle metabolic process c1=0.00989
GO:0006560 proline metabolic process c1=0.0141
GO:0065007 biological regulation
GO:0023052 signaling c2=0.000451
GO:0009987 cellular process c1=0.0382
GO:0050789 regulation of biological process
GO:0023046 signaling process c1=0.0351 | c2=0.000406
GO:0051301 cell division c2=0.0277
GO:0007049 cell cycle
GO:0071103 DNA conformation change col1=0.000223 | col2=0
GO:0050794 regulation of cellular process
GO:0023060 signal transmission c1=0.0351 | c2=0.000406
GO:0000278 mitotic cell cycle c2=0.014
GO:0022402 cell cycle process c2=0.00562
GO:0006323 DNA packaging col1=8.25e-05 | col2=0
GO:0007165 signal transduction c2=0.00234
GO:0022403 cell cycle phase c2=0.00741
GO:0000279 M phase c2=0.00933
GO:0016043 cellular component organization c2=3.86e-05
GO:0044085 cellular component biogenesis col1=0.0013 | col2=2.26e-07
GO:0006996 organelle organization col1=0.0102 | col2=2.46e-11
GO:0022613 ribonucleoprotein complex biogenesis c1=0.0223
GO:0043933 macromolecular complex subunit organization col1=0.00452 | col2=1.73e-11
GO:0022607 cellular component assembly col1=0.0249 | col2=4.38e-10
GO:0051276 chromosome organization col1=0.000335 | col2=0
GO:0042254 ribosome biogenesis c1=0.025
GO:0034621 cellular macromolecular complex subunit organization col1=0.00199 | col2=1.14e-12
GO:0065003 macromolecular complex assembly col1=0.00785 | col2=1.59e-11
GO:0006325 chromatin organization col1=0.000233 | col2=0
GO:0034622 cellular macromolecular complex assembly col1=0.0036 | col2=8.49e-13
GO:0006333 chromatin assembly or disassembly col1=6.74e-05 | col2=0
GO:0034728 nucleosome organization col1=6.59e-05 | col2=0
GO:0031497 chromatin assembly col1=6.59e-05 | col2=0
GO:0065004 protein-DNA complex assembly col1=6.59e-05 | col2=0
GO:0006334 nucleosome assembly col1=6.59e-05 | col2=0
GO:0050896 response to stimulus c1=0.0007
GO:0009628 response to abiotic stimulus
GO:0006950 response to stress c1=8.06e-05
GO:0009607 response to biotic stimulus c1=0.000619
GO:0009605 response to external stimulus c1=0.0363
GO:0009266 response to temperature stimulus c1=0.00474
GO:0006952 defense response c1=0.00199
GO:0009611 response to wounding c1=0.00599
GO:0009408 response to heat c1=0.0107
GO:0009409 response to cold c1=0.0132

B

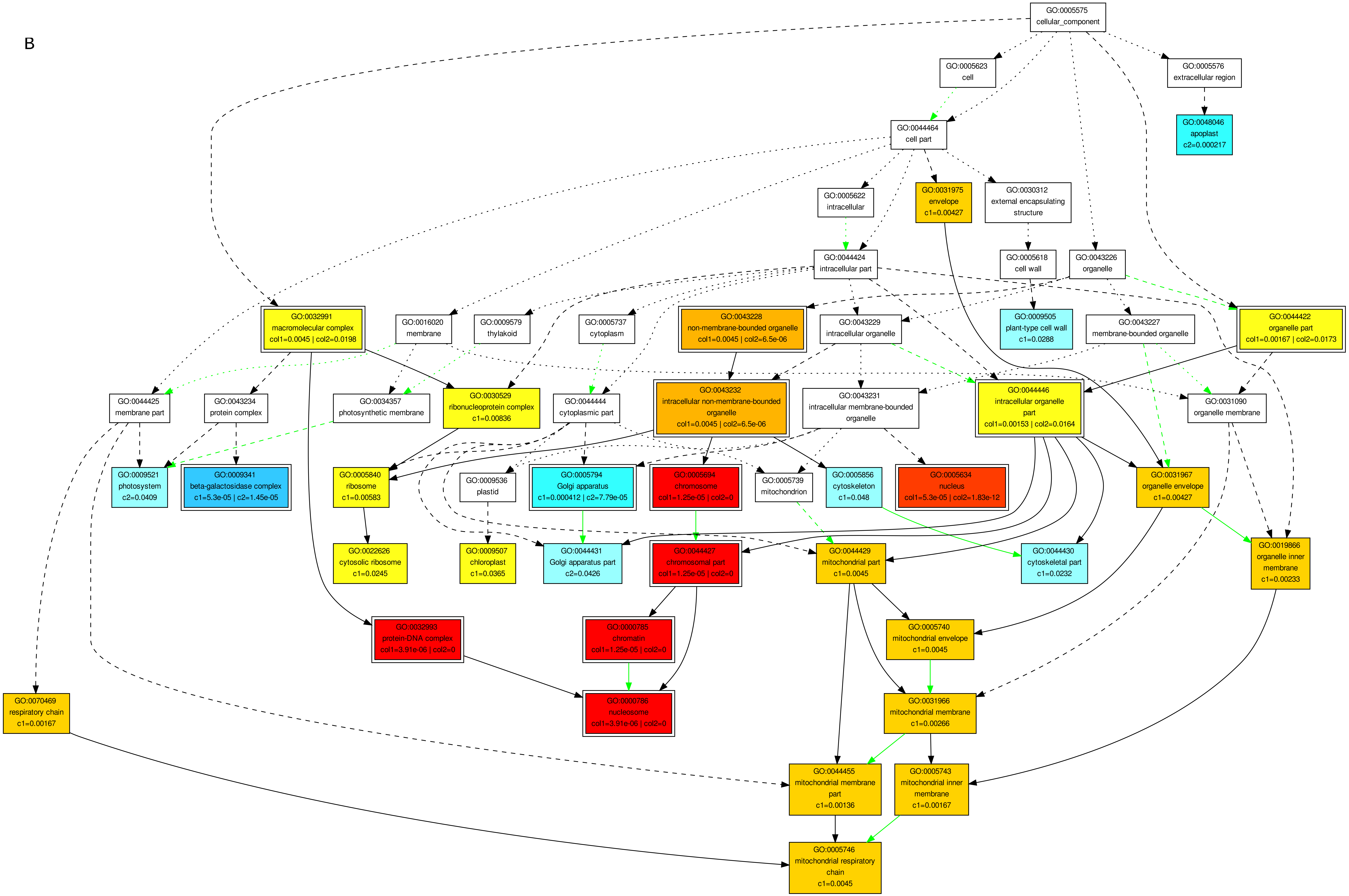

GO:0005575 cellular_component
GO:0005623 cell
GO:0005576 extracellular region
GO:0044464 cell part
GO:0048046 apoplast c2=0.000217
GO:0005622 intracellular
GO:0031975 envelope c1=0.00427
GO:0030312 external encapsulating structure
GO:0044424 intracellular part
GO:0005618 cell wall
GO:0043226 organelle
GO:0032991 macromolecular complex col1=0.0045 | col2=0.0198
GO:0016020 membrane
GO:0009579 thylakoid
GO:0005737 cytoplasm
GO:0043228 non-membrane-bounded organelle col1=0.0045 | col2=6.5e-06
GO:0043229 intracellular organelle
GO:0009505 plant-type cell wall c1=0.0288
GO:0043227 membrane-bounded organelle
GO:0044422 organelle part col1=0.00167 | col2=0.0173
GO:0044425 membrane part
GO:0043234 protein complex
GO:0034357 photosynthetic membrane
GO:0030529 ribonucleoprotein complex c1=0.00836
GO:0044444 cytoplasmic part
GO:0043232 intracellular non-membrane-bounded organelle col1=0.0045 | col2=6.5e-06
GO:0043231 intracellular membrane-bounded organelle
GO:0044446 intracellular organelle part col1=0.00153 | col2=0.0164
GO:0031090 organelle membrane
GO:0009521 photosystem c2=0.0409
GO:0009341 beta-galactosidase complex c1=5.3e-05 | c2=1.45e-05
GO:0005840 ribosome c1=0.00583
GO:0009536 plastid
GO:0005794 Golgi apparatus c1=0.000412 | c2=7.79e-05
GO:0005694 chromosome col1=1.25e-05 | col2=0
GO:0005739 mitochondrion
GO:0005856 cytoskeleton c1=0.048
GO:0005634 nucleus col1=5.3e-05 | col2=1.83e-12
GO:0031967 organelle envelope c1=0.00427
GO:0022626 cytosolic ribosome c1=0.0245
GO:0009507 chloroplast c1=0.0365
GO:0044431 Golgi apparatus part c2=0.0426
GO:0044427 chromosomal part col1=1.25e-05 | col2=0
GO:0044429 mitochondrial part c1=0.0045
GO:0044430 cytoskeletal part c1=0.0232
GO:0019866 organelle inner membrane c1=0.00233
GO:0032993 protein-DNA complex col1=3.91e-06 | col2=0
GO:0000785 chromatin col1=1.25e-05 | col2=0
GO:0005740 mitochondrial envelope c1=0.0045
GO:0070469 respiratory chain c1=0.00167
GO:0000786 nucleosome col1=3.91e-06 | col2=0
GO:0031966 mitochondrial membrane c1=0.00266
GO:0044455 mitochondrial membrane part c1=0.00136
GO:0005743 mitochondrial inner membrane c1=0.00167
GO:0005746 mitochondrial respiratory chain c1=0.0045

C

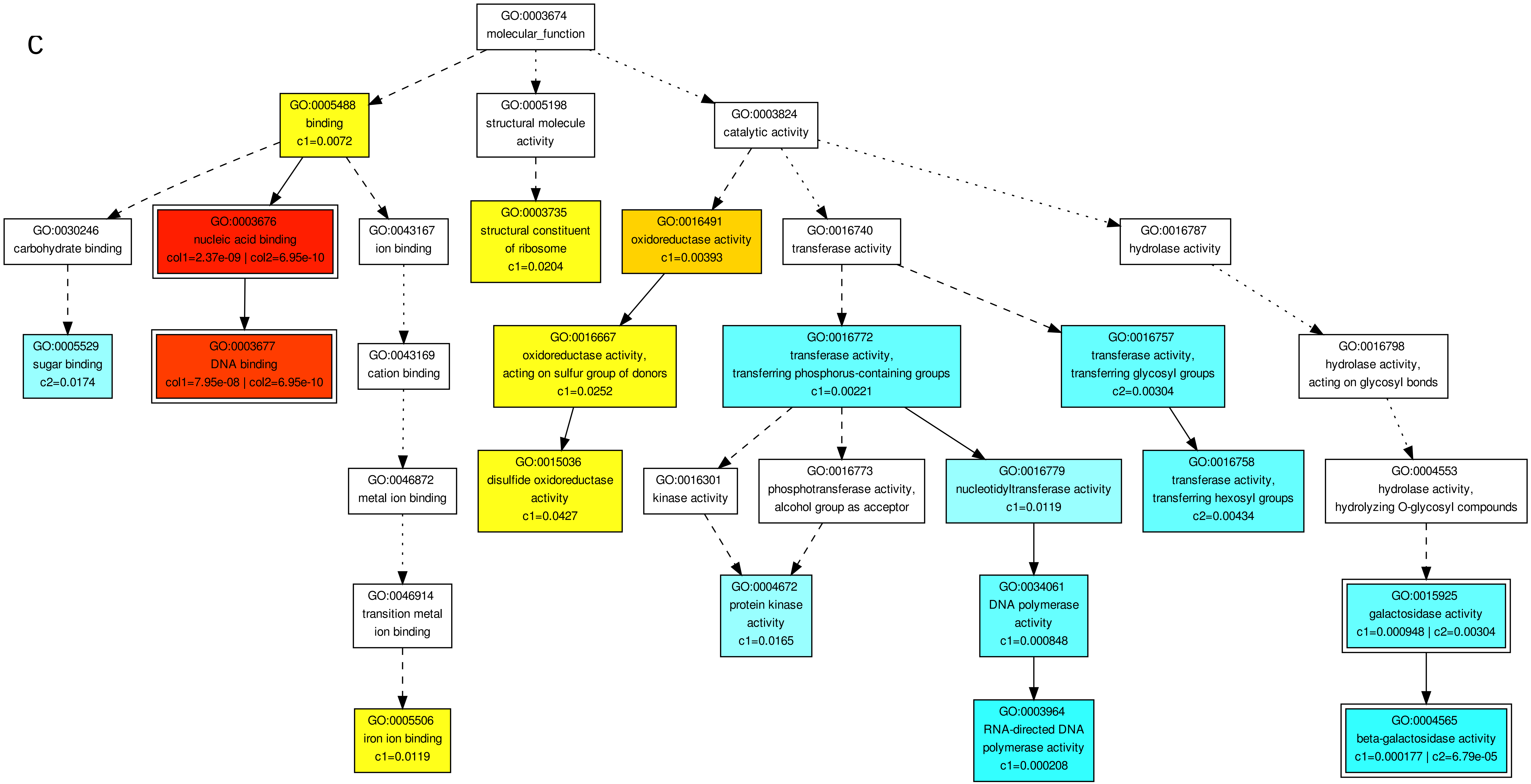
GO:0003674
molecular_function
GO:0005488
binding
c1=0.0072
GO:0005198
structural molecule
activity
GO:0003824
catalytic activity
GO:0030246
carbohydrate binding
GO:0003676
nucleic acid binding
col1=2.37e-09 | col2=6.95e-10
GO:0043167
ion binding
GO:0003735
structural constituent
of ribosome
c1=0.0204
GO:0016491
oxidoreductase activity
c1=0.00393
GO:0016740
transferase activity
GO:0016787
hydrolase activity
GO:0005529
sugar binding
c2=0.0174
GO:0003677
DNA binding
col1=7.95e-08 | col2=6.95e-10
GO:0043169
cation binding
GO:0016667
oxidoreductase activity,
acting on sulfur group of donors
c1=0.0252
GO:0016772
transferase activity,
transferring phosphorus-containing groups
c1=0.00221
GO:0016757
transferase activity,
transferring glycosyl groups
c2=0.00304
GO:0016798
hydrolase activity,
acting on glycosyl bonds
GO:0046872
metal ion binding
GO:0015036
disulfide oxidoreductase
activity
c1=0.0427
GO:0016301
kinase activity
GO:0016773
phosphotransferase activity,
alcohol group as acceptor
GO:0016779
nucleotidyltransferase activity
c1=0.0119
GO:0016758
transferase activity,
transferring hexosyl groups
c2=0.00434
GO:0004553
hydrolase activity,
hydrolyzing O-glycosyl compounds
GO:0046914
transition metal
ion binding
GO:0004672
protein kinase
activity
c1=0.0165
GO:0034061
DNA polymerase
activity
c1=0.000848
GO:0015925
galactosidase activity
c1=0.000948 | c2=0.00304
GO:0005506
iron ion binding
c1=0.0119
GO:0003964
RNA-directed DNA
polymerase activity
c1=0.000208
GO:0004565
beta-galactosidase activity
c1=0.000177 | c2=6.79e-05
